# Translational 3D *in vitro* models for immunotherapy testing: from reconstituted organoid co-culture assays to autologous *ex vivo* patient tissues

**DOI:** 10.3389/fimmu.2026.1721016

**Published:** 2026-05-08

**Authors:** Saskia de Man, Ezgi Kaya-Aksoy, Tomas Veenendaal, Niels Meesters, Sergei Chavez Abiega, Xuefei Yan, Michelle Kop, Luca Gandini, Jolie Flach, Jiawei Meng, Meng Zhang, Valeria Teodosieva, Jarmil Hanrath, Chiara Foini, Talita Stessuk, Emma Spanjaard, Richard Shih, Xiaolong Tu, Jun Zhou, Peng Wang, Nataliia Beztsinna, Gera Goverse, Ludovic Bourré, Sander Basten, Marrit Putker

**Affiliations:** 1 Crown Bioscience NL, Leiden, Netherlands; 2 Crown Bioscience CN, Taicang, Jiangsu, China; 3 Crown Bioscience Inc., San Diego, CA, United States

**Keywords:** *ex vivo* patient tissue, high content imaging, immunotherapy, organoid, preclinical drug testing

## Abstract

The lack of patient-relevant, clinically predictive models for early (immuno)-oncology (IO) drug discovery is a significant challenge for drug developers. Advances in three-dimensional (3D) cell culture technologies are narrowing the gap for preclinical drug testing, capturing cell–cell and cell–matrix interactions, extracellular matrix components, and spatial organization absent in 2D systems and crucial in modeling immunotherapy efficacy. Moreover, these advanced systems more accurately reflect tumor genetics, antigen expression levels, tumor heterogeneity, and critically, have proven to be highly predictive of clinical responses. Here, we present two novel high-content imaging-based 3D *in vitro* platforms for immune-modulating drug screening: a reconstituted organoid-based model that recreates key aspects of the tumor microenvironment under controlled conditions, and a native *ex vivo* patient tissue platform that retains the original components of the microenvironment, including immune cell composition and tumor heterogeneity, enabling comparative testing of immunotherapies in an autologous setting. Both methods were evaluated using multiple therapeutic modalities, comparing their ability to capture relevant immune responses. By outlining their strengths, limitations, and translational potential, this work highlights how advanced 3D *in vitro* models can accelerate development of effective immune-modulating therapies, aid in biomarker identifications, and inform personalized treatment strategies.

## Introduction

Immuno-therapies, most notably immune checkpoint inhibitors (ICI) targeting proteins like PD-1/PD-L1, have transformed oncology by harnessing the body’s own T cells to attack tumor cells (1, 2). While these breakthroughs have yielded remarkable and durable responses in some cancer patients, the majority of patients fail to benefit, underscoring the complexity of the tumor immune landscape and the need for predictive biomarkers of response (3, 4). Recognition of the immense potential of immunotherapies for oncology purposes has led to a surge in the identification of novel oncology drug targets and the development of diverse therapeutics (5). Despite these innovations, preclinical and clinical tests in solid cancers have achieved limited success, with durable response rates averaging <15% across indications (3). A deeper understanding of tumor immune landscapes, patient’s related responses, and strategies to modulate these responses are required to improve clinical outcomes.

A major challenge for early (immuno)-oncology drug discovery is the lack of patient-relevant, clinically predictive models (4, 6). Although traditional 2D cell line culture systems are easily scalable and come with comprehensive historical molecular and pharmacological characterization, long-term 2D growth on plastic drives cells to diverge from their native state, leading to genetic alterations, activation of stress pathways, and the loss of physiologically relevant microenvironmental features such as cell–cell and cell–matrix interactions (7). As a result, this limits their physiological relevance, translational predictivity, and holds back development of highly efficient therapeutics.

In contrast, three-dimensional (3D) culture systems provide a biologically more relevant context by supporting among others physiologically critical interactions between polarized cells and their extracellular environment (8), that are closer to *in vivo* tumor conditions, impacting cellular signaling, proliferation, differentiation, survival, and pharmacological responses (9–12). These key features enhance the predictive capacity of 3D systems, guiding more informed decision-making in early drug development phases.

Among 3D models, patient-derived tumor organoids (PDOs) have emerged as highly representative and versatile *in vitro* models (11, 13, 14). Organoids are multicellular, self-organizing microstructures generated from adult cells extracted directly from healthy or diseased tissue like patient tumors (PDO) or patient-derived xenograft (PDX) models (PDXOs) (15, 16). They maintain the structural architecture, genetic heterogeneity, and molecular phenotypes of the parental tumors, exhibit robust *in vitro* expansion, and can be biobanked to capture patient population diversity (14, 16–19). Importantly, several clinical studies have exemplified the clinical translatability of organoid studies, with high correlations between organoid and patient responses (11, 18, 20–22). This predictability, combined with large organoid biobanks, enables comprehensive and reproducible testing across heterogeneous tumor types, and allows organoids to serve as reliable “patient avatars” for predictive pharmacological screening (23).

**Figure 1:**
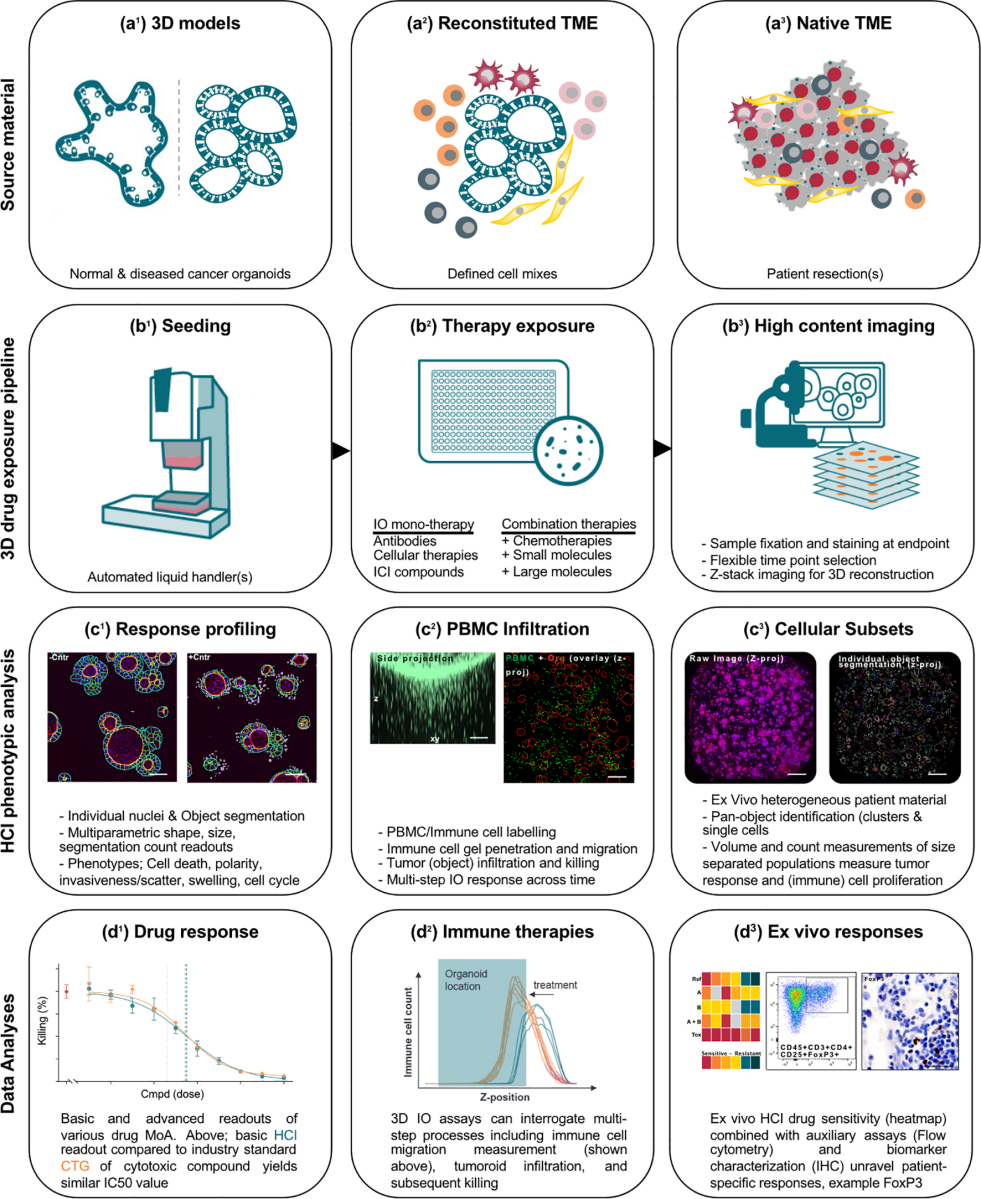
Schematic overview of HCI-based assays for immuno-oncology applications. **(a)** In the previously established organoid screening platform, source material can be normal or diseased cancer organoids alone in an extracellular matrix resembling hydrogel (a^1^). To modify this into an IO relevant setting, a reconstituted tumor TME is generated by mixing organoids with typical TME components such as PBMCs, specifically isolated immune cells, and/or stroma components such as fibroblasts (a^2^). Alternatively, fresh tumor resection material from cancer patients containing a unique patient-specific TME composition is used as input, a methodology described as the *Ex vivo* patient tissue (EVPT) assay (a^3^). **(b)** A 3D drug exposure pipeline handles the source material which is seeded in 3D into multi-well screening plates using liquid handling robotics (b^1^) by embedding in a protein-rich hydrogel extracellular scaffold molecules such as laminin and collagen. Seeding density is >50–300 tumoroids or >0.01 mm^3^ microtissue per well. A broad collection of biologics can be co-incubated, including small molecules, (lipid) nanoparticles, antibodies and antibody-derivatives, viruses, and cellular therapies (b^2^). At the experimental endpoint, screening plates are fixed, stained with fluorescent marker molecules, followed by acquisition of image-stacks per well (b^3^). **(c)** High content imaging (HCI) phenotypic analyses where source images are converted to a 3D image reconstruction via a proprietary in-house developed segmentation-based image analyses platform (Ominer) from which multiparametric readouts can be extracted that are used to capture responses, such as basic cell/object shape and numerical readouts, as well as cell viability and cytotoxic readouts (c^1^), immune cell migration and tumor infiltration (c^2^), and resolve complex tissues responses to identify distinct subset responses (c^3^). **(d)** Imaging features or measurements are analyzed and interpreted by biologists and translated into data reports. Readout parameters allude to tumor dimensions and cell survival (d^1^), immune cell dynamics and efficacy in tumor killing (d^2^), and *ex vivo* tissue responses (d^3^). Combined with auxiliary assays and additional sample characterization such as FoxP3 staining to identify Tregs, a full picture emerges of tissue responsiveness. Scale bars represent in c^1^, d^3^ 50 µm, c^2^, c^3^ 500 µm.

Multiple applications of organoids in immuno-oncological drug testing have previously been described (24–26). Co-culture systems have proven feasible and translatable; however, scalability and reproducibility are limiting the application in (early) drug development pipelines. Therefore, we built complexity into a streamlined 384-well plate-, high-content imaging (HCI)-based platform that allows insightful, large throughput, reproducible and translatable testing of IO therapeutics, designed for industrial deployment (**Figure 1**).

Notwithstanding the scalable and flexible assay setup, reconstituted assays are intrinsically limited by the set level of cellular complexity and genetic compatibility of input materials. Certain therapeutic agents, such as antibody and cellular therapy-based immunomodulatory therapies and some cancer vaccines may require either an in-depth characterization for biologic compatibility in a reconstituted assay, or simply require more cellular complexity. This includes therapies benefitting from an autologous immune system in the case of MHC-dependent tumor killing post-ICI therapy, those targeting phagocytic checkpoints, or whose target is present on multiple cell populations (27) and where the patient-specific interplay between tumor cells, immune infiltrates (e.g. exhausted, activated, suppressive populations), and stromal components, requires a full tumor microenvironment (TME) encompassing setting. Similarly, late-stage drug development programs can benefit from testing in an as-near clinical setting context where the cellular complexity is present to truly interrogate drug efficacy.

*Ex vivo* drug testing on primary tumors has been shown to be predictive of patient responses, but assays described assays are complex and require expertise to be implemented into preclinical pipelines (28–30). To allow for commercial testing of approved and preclinical IO therapeutics in an autologous setting, a relatively scalable and robust *ex vivo* patient tissue (EVPT) platform utilizing primary tumor tissues was developed. Created in parallel with the reconstituted platform, it is leveraging the same HCI-based technological framework and advances.

## Results

### Development and validation of a reconstituted and scalable immuno-oncology drug testing platform

To reconstitute the TME in a fully controlled, scalable, and reproducible assay, patient-derived organoids were seeded in a dense extracellular matrix mix (800-1200 µm) in 384-well plates in an automated fashion. After 3–5 days of maturation, pre-labeled PBMCs from healthy donors were seeded on top, as well as immuno-oncology therapeutics, and co-culture experiments were fixed and stained at several timepoints (ranging from 2–7 days), while medium was stored for subsequent cytokine analysis. The allogenic 3D co-cultures were imaged in a 3D stack with a confocal high content microscope to visualize the interaction and positioning of organoids and immune cells, and automated image analysis was performed using proprietary analysis software.

**Figure 2:**
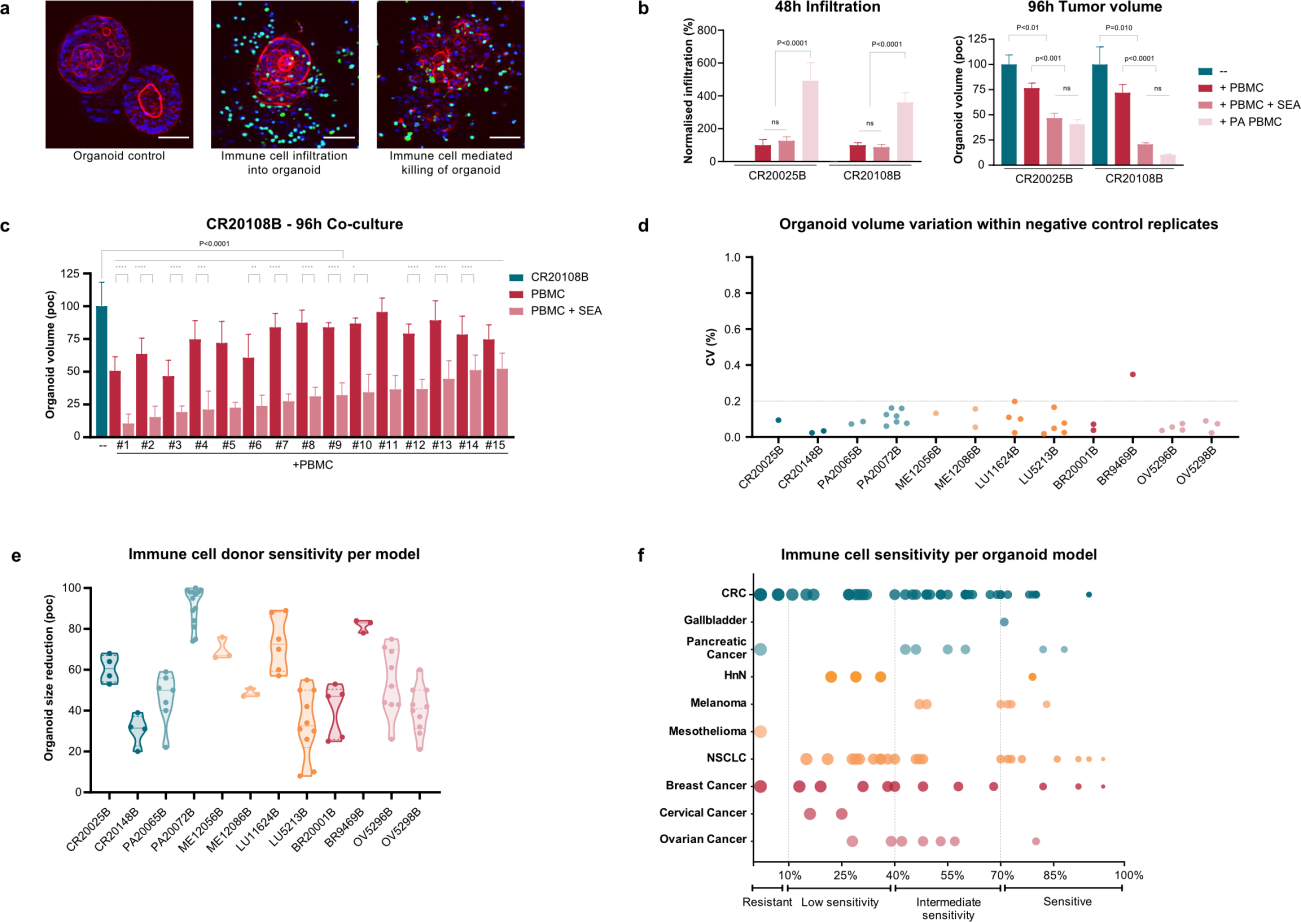
Reconstituted organoid-PBMC co-culture assay shows various levels of immune infiltration and killing. **(a)** High content images of CRC organoid co-cultures showing nuclei (blue), Actin (red) and PBMCs (green) in organoid only, immune cell infiltration and immune cell mediated killing conditions. **(b)** Two CRC organoid models were co-cultured with naïve or pre-activated (PA) (SEA) PBMCs and activated in co-culture. After 48h of co-culture, immune cell infiltration was measured and normalized to naïve PBMCs. Total organoid volumes, normalized shown as percentage of control (poc), were analyzed after 96h. **(c)** One CRC organoid model co-cultured with PBMC of 15 different donors analyzed for its total volume after 96h. Organoids only is compared with SEA treated PBMCs. Per PBMC donor, with and without SEA are compared. **(d)** Scatter plot showing 12 organoid models from CRC (CR), pancreatic (PA), melanoma (ME), lung (LU), breast (BR) and ovarian (OV) which were analyzed for CV%, defined as the ratio of the standard deviation to the mean, in the negative control condition. Each dot represents one set of quadruplicates tested, multiple dots per model represent different experiments. **(e)** Violin plot demonstrating the variation in organoid sensitivity toward immune cell mediated killing in the same 12 models as shown in **(d)**, measured by organoid size reduction as percentage of control. Each dot represents one set of replicates, coming from either multiple experiments or multiple donors. **(f)** Plot demonstrating the organoid sensitivity toward immune cell-mediated killing within >100 models from 10 indications ranked from resistant to sensitive models. Set of n=4 technical replicates with mean and SD shown. Statistical analysis used for 2b is two-way ANOVA, followed by multiple comparisons using Tukey’s methodology, 2c is Ordinary One-way ANOVA, followed by *post-hoc* multiple comparison test using Tukey’s methodology. **** P<0.0001, *** P<0.005, ** P<0.01, * P<0.05. Scale bars in 2a represent 100 µm.

The pipeline was initially evaluated using two colorectal cancer (CRC) PDO models. Immune cell infiltration into the organoids was detected, while activation with super-antigen SEA, a strong immune stimulatory agent, of the immune cells led to killing of the organoids (**Figure 2a**). Quantitative image analysis detected enhanced infiltration of SEA pre-activated immune cells after 48h, while organoid killing was measured by reduced total organoid volume after 96h (**Figure 2b**). Reductions in the total organoid volume resulted from both a reduction in organoid size and loss of organoid objects, and also corresponded to increased cleaved Caspase-3 positive nuclei in IF staining (data not shown). Here, both CRC models demonstrated an assay window that allowed testing of immunotherapies with this specific PBMC donor.

To evaluate the potential role of PBMC donor variability in allogenic responses, a donor screen including a total of 15 healthy donors was performed (**Figure 2c**). Although an expected donor-to-donor variability was observed, overall consistency was obtained in the immune cell-mediated killing, showing a ~20% reduction of total organoid volume across addition of naive PBMCs from various donors, which was further enhanced to ~50-80% upon activation with SEA.

Assay robustness was further assessed for 12 organoid models across multiple donors and experiments, based on quantitative performance within four replicates, where the coefficient of variation (CV%) was used to evaluate the precision of organoid seeding (**Figure 2d**). All models showed low percentages of variation (<0.35) indicating high consistency per run and across multiple runs. Additionally, the same models were evaluated for their immune cell-mediated killing with multiple donors in different experiments (**Figure 2e**). Similar to what was found in **Figure 2c**, organoid killing was mostly determined by the organoid model, rather than by experimental and donor variation.

Next, scalability of the co-culture assay was evaluated, by assessing the sensitivity of a total of more than 100 organoid models from 10 different indications to killing by activated PBMCs (**Figure 2f**). For most indications in which multiple organoid models were tested, a spectrum of responses to immune cell–mediated killing was observed, ranging from resistant to highly sensitive models. For some indications the sensitivity variation observed was gradual (e.g., CRC and Breast cancer), whereas for other indications a clear separation was observed between sensitive and resistant models.

### Applying organoid-PBMC co-culture assays to test different IO modalities

**Figure 3:**
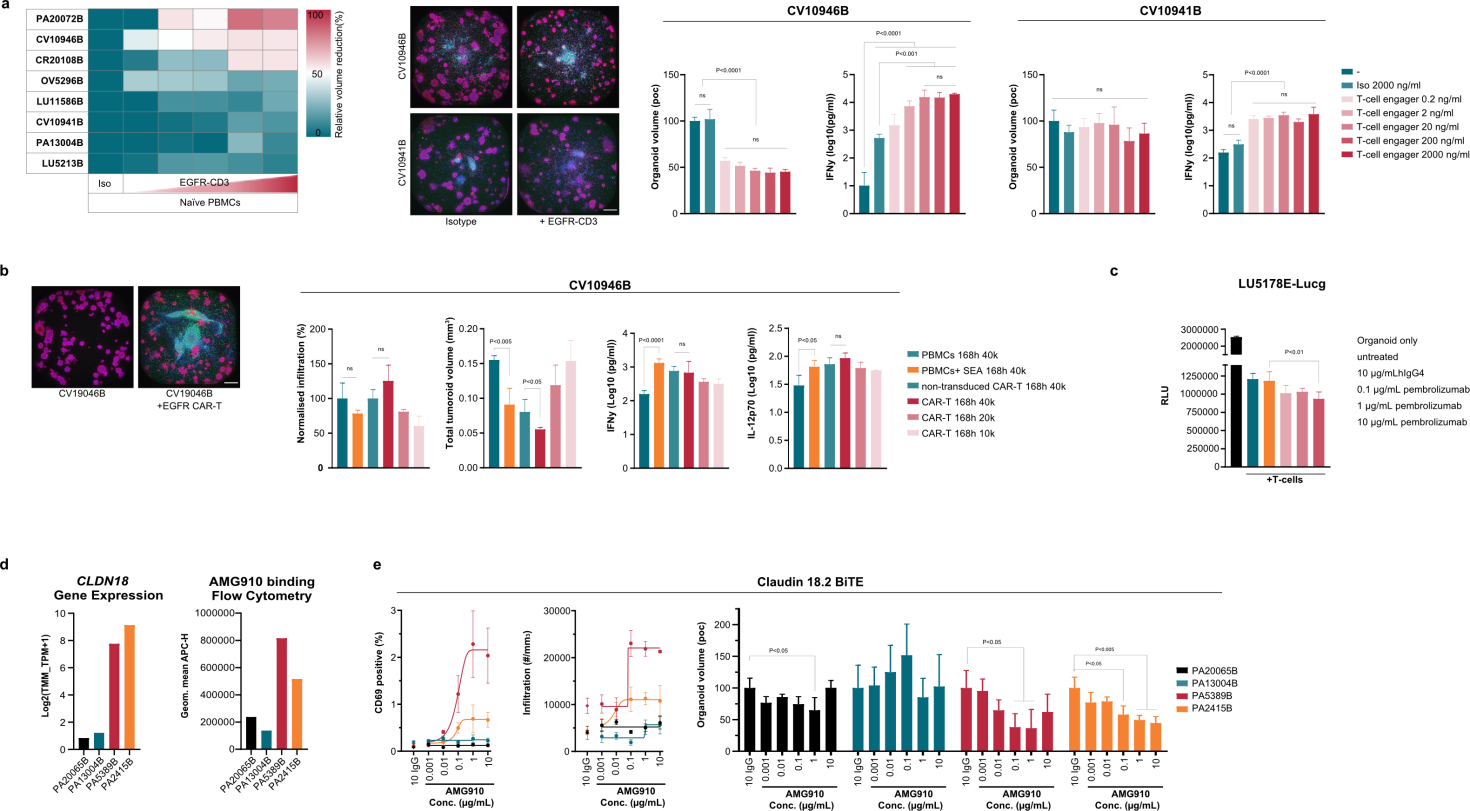
Co-culture assay allows versatile IO drug testing. **(a)** Heatmap showing reduced organoid volume upon treatment with CD3-EGFR (dosed between 2000 and 0.20 ng/ml) compared to the isotype control within eight organoid models. Representative images of two cervical models are presented, showing effect of isotype and CD3-EGFR exposure. Bar graphs show image quantification results: normalized organoid volume and IFN-γ levels measured in the supernatant of the co-cultures. **(b)** Organoid model CV19046B was co-cultured with EGFR targeting and non-transduced CAR T cells showing images, immune cell infiltration in the organoids, total organoid volume and cytokine levels of IFN-γ and IL-12p70 measured in the supernatants. **(c)** Luminescence of lung organoid model LU5178-Lucg co-cultured with pre-activated T cells upon treatment with Pembrolizumab or the isotype control. Set of n=4 technical replicates with mean and SD shown. **(d)** Left: Claudin 18.2 (*CLDN18*) gene expression in four Pancreatic cancer (PA) models as quantified by bulk RNAseq analysis. Right: Claudin 18.2 protein expression determined by FACS analysis. Per condition, n=1; minimum of 10.000 events counted per condition). **(e)** PA organoid models were co-cultured with PBMCs and treated with 0.001-10 µg/mL AMG910 or 10 µg hIgG control. Graphs show the percentage of CD69^+^ PBMCs (left), PBMC infiltration in the organoids (middle) and total organoid volume normalized to the hIgG control (right). Statistical analysis used for 3a, 3e is ordinary One-way ANOVA, followed by *post-hoc* multiple comparison test using Tukey’s methodology. For 3b means are compared using unpaired t-test. For 3c a Two-Way ANOVA was used, followed by multiple comparisons using Dunnett methodology. ns, not significant. Scale bars in 3a and 3b represent 500 µm.

The baseline characterization reported in **Figure 2f**, in combination with deep NGS characterization of all reported organoid models, allows selection of models for follow up studies based on their predicted immune-sensitivity and their RNA expression profiles. Given the reconstituted co-culture platform is suitable for standardized and scalable immunotherapy evaluation, its utility was next assessed in characterizing different IO modalities. First, the activity of bispecific T cell engagers (BiTEs) was evaluated. Eight different organoid models (selected for their EGFR expression levels) from four different indications were co-cultured with naive PBMCs and a dose range of EGFR-CD3 BiTE as well as the BSA-CD3 isotype control. Based on the total organoid volume normalized to the isotype control, three out of the eight models showed a dose-dependent enhanced killing upon treatment with the BiTE (**Figure 3a**). Focusing on two cervical organoid models, images indeed confirmed this killing effect upon treatment with the EGFR-CD3 in model CV10946B, while no effects were detected with model CV10941B. Interestingly, when the supernatants collected from these co-cultures were analyzed for cytokine release, similar increased IFN-γ levels were observed in co-cultures of both cervical organoid models (**Figure 3a**), suggesting that PBMC activation does not always correlate to cancer cell killing, and that complementary HCI analysis is required to assess the extent of the effect of immune cell activation on the organoids.

Next, the reconstituted organoid co-culture model was applied to testing further immunotherapeutic modalities, such as cellular therapies and immune checkpoint inhibitors. First, EGFR targeting CAR-T cell efficacy was tested in the same cervical organoid model that also demonstrated sensitivity upon treatment with the EGFR-CD3 BiTE. Targeting the same tumor antigen, these CAR-T cells indeed also induced organoid killing (**Figure 3b**). EGFR CAR-T cells exhibited greater infiltration into the organoids compared to non-transduced CAR-T cell controls, which was accompanied by a significant reduction in total organoid volume. Analysis of co-culture supernatants revealed that both non-transduced and antigen-specific CAR T cells produced high levels of the inflammatory cytokines IFN-γ and IL-12, consistent with the activation with CD3/CD28 beads during the expansion of both cell types. The lack of difference between cytokine production further supports that combining HCI with cytokine measurements is required to accurately distinguish specific effects of immunotherapeutic interventions.

The application of the organoid-immune cell co-culture setup for testing immune checkpoint inhibitor Pembrolizumab (Pembro) was assessed in a luciferase-based assay. Treatment with the highest dose of Pembro (10 µg/ml) enhanced the killing of lung tumor organoid model LU5178-Lucg in co-cultures with pre-activated T cells (**Figure 3c**). The observed effect was donor-dependent (data not shown), indicating that multi-donor testing is necessary for robust ICI evaluation.

Finally, the correlation between biomarker expression and efficacy was assessed. Claudin 18.2 is an interesting target for immune therapy targeting, due to its restricted expression in healthy tissue and frequent overexpression in solid tumors (31). Based on the available NGS data, four pancreatic organoid models were selected for respective high (2) and low (2) *CLDN18* mRNA expression. Correlation with protein expression was confirmed using flow cytometry (**Figure 3d**). The organoids were co-cultured with naïve PBMCs and treated with AMG910, a half-life extended Claudin 18.2-targeting BiTE (32). Specific activation of PBMCs, measured via CD69 staining, as well as increased subsequent tumor infiltration and tumor-killing were observed only for the two high expressing Claudin 18 models (**Figure 3e**), validating the correlation between TAA expression and immune-cell activation and immune-mediated killing.

### Application of *ex vivo* patient tissues in HCI platform reveals patient-specific ICI responses in autologous setting

Whereas the reconstituted assay proved scalable, controllable, and amendable to include various key players of the TME, it inherently lacks autologous TME components. To allow therapy testing in presence of native, autologous cells and factors from the microenvironment, and thereby to complement the reconstituted assay, an *ex vivo* patient tissue (EVPT) assay was developed. Here, fresh patient resection material (treatment-naïve) was received in the laboratory within 24 hours post-surgery, cleared of necrotic regions and fat deposits, and then mechanically disrupted with minimal processing. The resulting heterogeneous mix of <200 µm small tumor clusters (tumoroids) and single cells making up the entire TME was used as a direct source for the functional assay, using a similar liquid handling automation and HCI screening setup as described before (**Figure 1**). A stringent quality control excluded low-quality samples when viability of the material was low and processed tissues were lacking heterogeneity of size. Depending on the pathology and cancer indication ~<50% of tissues were eligible for the assay.

**Figure 4:**
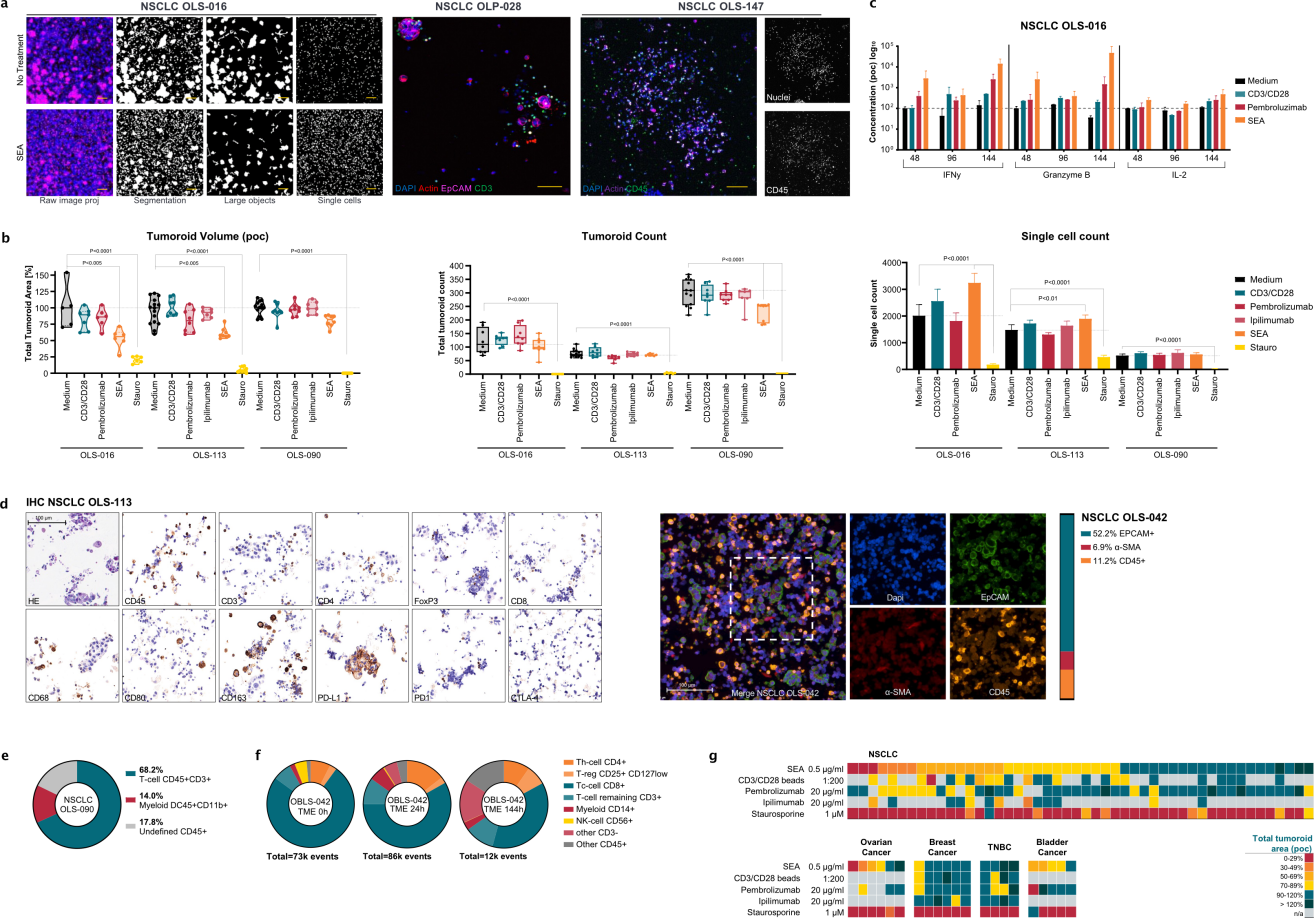
ICI testing in *Ex Vivo* Patient Tissue assay in native TME environment. **(a)** Left panel shows cropped images from sample OLS-016 shown as a projection from an image stack, part of a single well. Raw images of nuclei stained with Hoechst33342 and actin cytoskeleton with rhodamine-Phalloidin. Object segmentation output after Ominer processing, separated for large object and single cells. Middle and right panels show high resolution images of samples OLP-028 and OLS-147 immuno-stained with EpCAM and CD3, and CD45 antibodies, confirming presence of both multicellular and single tumor cells as well as immune infiltrated cells in the resection samples present after 6 days culture. **(b)** Quantified HCI readout of untreated negative control (medium), immuno-stimulatory controls CD3/CD28 Dynabeads and SEA, immune checkpoint inhibiting (ICI) Pembrolizumab and Ipilimumab, and toxicity control Staurosporin in NSCLC samples OLS-016, OLS-113, OLS-090. Normalized, as percentage of control (poc), tumoroid size (median) shown in a violin plot, object count (median) shown in a box plot, single cell count (mean) shown in a bar chart. n=8 replicates per condition **(c)** Cytokine analyses in pooled supernatants per treatment condition collected from OLS-016 assay plate for analytes IFN-γ, Granzyme B, and IL-2. Averages of two pooled sets of quadruplicates with standard deviation shown, no statistics shown. **(d)** Single DAB staining and Multiplex IF (mIF) on FFPE embedded tumor fragments from patient-derived NSCLC samples. Single staining for epithelial marker Pan-CK, TME and immune cell and biomarkers α-SMA, CD45, CD3, CD4, CD8, CD68, CD80, CD163, FoxP3, PD-L1, PD1, CTLA-4. mIF for epithelial marker EpCAM, and TME markers α-SMA and CD45. Quantification on the stacked bar shows the representative presence of each subpopulation in the mIF sample. **(e)** Flow cytometry analyses of the immune populations (CD45^+^) of sample OLS-090 identifies most cells as basic T cells (CD3^+^) next to myeloid cells (CD11b^+^) presented in a donut plot. **(f)** A more extensive flow cytometry panel tracks subsets of immune cells at baseline and timepoints 24h and 144h represented in donut plots. Overall, relative proportions of cells remain stable in the first day, whereas after 144h fewer cells could be recovered from the sample, of which the CD8^+^ population was progressively less present. **(g)** Heatmaps of n=49 NSCLC, n=6 Ovarian cancer, n=10 Breast cancer, and n=5 Bladder cancer *ex vivo* samples’ responses to controls and immuno-stimulatory and ICI compounds in EVPT platform. Total tumoroid area was normalized as % of medium control for every sample. Shown is the median value from 8 technical replicate wells. Statistical analysis used for 4b is Ordinary One-way ANOVA, followed by *post-hoc* multiple comparison test using Tukey’s methodology. Scale bar in 4a segmentations (left) represents 200 µm in IF 100 µm (middle, right), in 4d 100 µm.

HCI analyses was used to distinguish tumor clusters from single immune cells based on size, as shown in **Figure 4a**. Immune-staining in-gel with tumor or immune markers confirms presence of EpCAM^+^ multicellular tumor clusters and cells, as well as CD3^+^ or CD45^+^ infiltrated immune cells. As proof of concept, non-small lung cancer (NSCLC) samples were prepared and treated with SEA, CD3/CD28 beads, PD1-inhibiting antibody pembrolizumab, CTLA-4 inhibiting antibody ipilimumab, or Staurosporin (Stauro; anti-fungal toxin, expected to kill all cells).

Quantitative image analysis showed that, compared to control conditions, the cumulative size of the measured large multicellular clusters decreased in response to SEA to various degrees in the samples (**Figure 4b**, left). To ensure a proper assay window for measuring this volumetric reduction of tumor clusters used as proxy for therapeutic response, a minimal threshold of at least 50 multicellular objects per well with a cumulative size of at least 0.01 mm^3^ in the control condition was set. Wells and samples that did not reach this threshold were QC failed. Additional parameters quantified were the tumoroid count, which was moderately affected in some samples (**Figure 4b**, middle panel), and proliferation of the single cell (containing immune cells) fraction, where increases were observed in OLS-016 and OLS-113 samples (**Figure 4b**, right panel). Upon stimulation with CD3/CD28 beads, a similar response, albeit more modest compared to SEA, was observed in OLS-016. Lastly, accurate assessment of ICI therapy using Pembrolizumab revealed tumor-reducing efficacy of this antibody within the complex and physiologically relevant TME in OLS-016. Immune cell activation in these samples was confirmed through cytokine analyses of the assay supernatant, where relevant cytokines such as IFN-γ, Granzyme B, and IL-2 (33) were found elevated over time in relevant treatment conditions compared to medium controls (**Figure 4c**). In sample OLS-113 there also was a pembrolizumab response measurable in the tumoroid volume and count measurements. Single antibody immune-histochemistry (IHC) staining of this sample confirmed presence of both PD-L1^+^ cells and PD1^+^ cells. Notably, the lack of an ipilimumab response in this sample corresponds with the absence of CTLA-4^+^ cells (**Figure 4d**). Sample OLS-090 shows no clear response to ICIs and can be categorized as a typical unresponsive sample to T cell specific therapies, possibly due to the relatively low number of immune cells present as defined by the measured single cell population.

To further characterize the complex TME present in the analyzed fresh tissue samples, a representative portion of the processed tissue, similar to what is seeded into the screening plates, was collected and immediately fixed for subsequent generation of Formalin-Fixed Paraffin-Embedded (FFPE) blocks. H&E slides were assessed as quality control measure, and multiplex immunofluorescence (mIF) or single IHC analysis was performed to identify relevant targets and/or cell types (**Figure 4d**). Illustrative of the rich native TME, epithelial tumor cells (EpCAM), CD45^+^ immune cells, and cancer associated fibroblasts (α-SMA) were identified. Similar results were found using alternative mIF panels using pan-cytokeratin and Vimentin as alternative tumor and stroma markers (not shown). Sample OLS-113 shows presence of various lymphocyte (CD3, CD4, CD8, FoxP3) markers, as well as myeloid markers (CD68, CD80, CD163) markers, alluding to a rich and diverse TME that is retained upon sample processing. For sample OLS-090, the TME was characterized using flow cytometry, which identified 31.3% of the single cells as CD45^+^, of which most are CD3^+^ T cells (**Figure 4e**).

Flow cytometry was further used to track sample composition longitudinally using a panel identifying T cell subsets, myeloid, and NK cells to characterize sample OBLS-042 from baseline to multiple timepoints upon incubation in hydrogel droplets. The number of recovered single CD45^+^ cells from the TME was stable between 21-26% across timepoints, the cell subsets are depicted in **Figure 4f**. The total number of cells measured after 144h is lower, where T cells, Tregs, and myeloid cells are proportionally more abundant, suggestive of better survival of these populations compared to known shorter lived cell types such as CD8^+^ cytotoxic T cells (34) and NK cells (35). Survival and proliferation cytokines are solely coming from the retained TME itself.

The performance of the EVPT assay was next evaluated in a larger cohort, focusing initially on resected non-small cell lung cancer (NSCLC) tissues. This indication was selected for its significant proportion of immunologically ‘hot’ tumors - relevant in context of cancer immunotherapy eligibility -, as well as its relevance as a disease area with significant unmet clinical need (36). Over 80 NSCLC tissues were tested for their immune modulation responses, of which 45 samples were exposed to the clinical standard-of-care ICIs Pembrolizumab or Ipilimumab. Markedly, a 20-25% response rate in tumor volume reduction to ICI was observed in these tested tissues (**Figure 4g**). These rates well align with reports of clinical outcomes across NSCLC immunotherapies (36). Additionally, when tissues were treated with the pan-immunostimulatory compound SEA, nearly half of the patient-samples exhibited a response. T cell stimulation using CD3/CD28 beads also proved to be effective in ~50% of the tested patient samples. Next, the assay was assessed for a wider range of tumor types, including ovarian cancer, breast cancer including TNBC, and bladder cancer, for the same immuno-oncology related compounds and therapies. The heatmaps in **Figure 4g** summarize the responses, highlighting sustained SEA and ICI responses across all tested tissues. Overall, breast cancer samples exhibited lower responsiveness to SEA, consistent with previous findings characterizing breast tumors as more immunologically “cold” compared to NSCLC.

### EVPT platform allows diverse IO therapy testing

**Figure 5:**
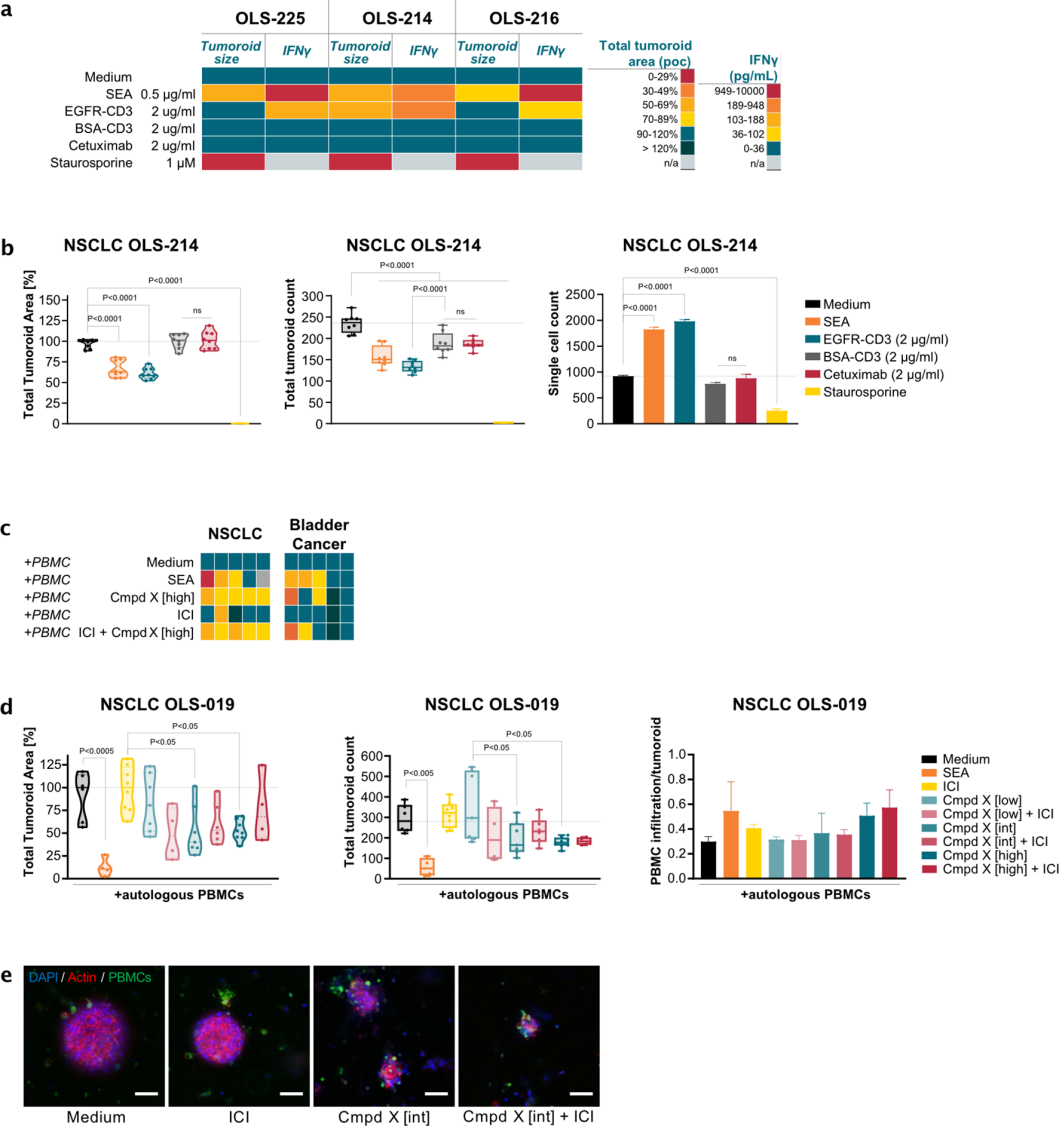
*Ex vivo* patient tissue assay allows IO drug efficacy assessment in native TME environment. **(a)** Heatmap of three NSCLC samples treated with T cell engager bi-specific antibody targeting CD3 and EGFR, or BSA in the control, Cetuximab, SEA, measured either with HCI analyses, or IFN-γ as a fast-readout for immune cell activation. **(b)** HCI measurements as described in (4B). **(c)** Heatmap of normalized tumoroid area measurements from HCI 10 primary samples embedded together with autologous PBMCs and exposed to medium, SEA, Pembrolizumab (ICI), experimental antibody compound X in low, intermediate (int), and high dose, and combination of the these with Pembrolizumab. **(d)** HCI measurements as described in (4b), and normalized infiltration measurement of autologous PBMCs into tumoroids. **(e)** Representative high-resolution images (cropped) of stained tumoroid objects (as in a) together with cell tracker labeled PBMCs. Set of n=8 technical replicates with mean or median and SD shown. Statistical analysis used for 5b, 5d is Ordinary One-way ANOVA, followed by *post-hoc* multiple comparison test using Tukey’s methodology. ns, not significant. Scale bar represents 50 µm.

Like the reconstituted assay, EVPT material allows testing of a variety of biologics, but in a more complex environment. **Figure 5a** shows three NSCLC samples tested for the same EGFR-CD3 bispecific antibody as shown in **Figure 3a**. Here, SEA induced immune-mediated tumor reduction in all three samples. One patient sample responded to the EGFR targeting BiTE, whereas EGFR-targeting Cetuximab or BSA-CD3 control bispecific failed to induce tumor reduction in any of the test samples. Immune activation in the EGFR-CD3 bispecific sensitive model was confirmed by increased IFN-γ secretion measured from assay supernatants. Although all samples showed elevated IFN-γ levels, only the sample where this was highest showed a measurable response using HCI, as shown in **Figure 5b** where both cumulative tumoroid volume and individual tumor cluster counts were decreased, and single cell counts elevated. In addition to resected tissue, blood draws from the same patients were collected, providing a source of patient-matched peripheral blood mononuclear cells (PBMCs) that could be isolated. Next, the assay was supplemented with a co-culture setup to provide an additional reservoir of autologous immune cells, potentially revealing responses in patient biopsy-derived tumoroids with low resident immune cells and allowing investigation of infiltration of autologous immune cells and interaction with tumor cells present in the tumoroid structure.

In **Figures 5c–e**, this approach was applied in the context of an undisclosed experimental antibody (compound X) targeting LLT1. LLT1 receptors are expressed in human tumor, interact with CD161 expressing NK cells and other immune cells to negatively regulate immune activation signals (37). By targeting the LLT1 receptor with blocking therapeutics, NK suppression can be relieved allowing an anti-tumor response from the innate immune system. This experimental class of treatments is often combined with traditional T cell targeted ICI. In **Figure 5c**, five tissues from NSCLC and bladder cancer patients were treated with control, SEA, or Pembrolizumab (ICI) in a co-culture setup with pre-labeled autologous PBMCs. SEA induced similar response rates across both tissues as in **Figure 4g**, and one ICI responder (20%) was identified in the NSCLC cohort. Experimental compound X alone and in combination with ICI showed measurable responses of tumor volume decrease in all NSCLC samples, and two Bladder cancer samples. More detailed measurements from the strongest SEA responsive NSCLC model in **Figure 5d**, showed multiple test doses of the experimental compound, both in monotreatment and in combination with a fixed ICI dose of 20 µg/ml Pembrolizumab. Whereas this model did not respond to ICI alone, nor to the lowest dose of the experimental compound, combination treatment with Compound X and ICI revealed significant reduction of tumoroid size and count. Concomitant increase in infiltration of labeled autologous PBMCs suggests anti-tumor effects of combination treatment. At higher doses, the monotreatment appeared dominant and no additive effect was observed in overall tumor volume. However, the number of tumor objects – heterogeneous in size – did show a robust decrease. Since the autologous supplemented immune cells were labeled with cell tracker dye, these cells could be tracked migrating into the tumor objects. Both in mono- and combination drug treatment with compound X this revealed a dose-dependent increase of immune cell infiltration, where highest infiltration levels aligned with SEA-induced effect. Representative images in **Figure 5e** support the tumor infiltrating and killing effect with the test compounds.

## Discussion

Drug attrition rates for anticancer agents remain high, with only ~5% of candidates reaching phase I achieving clinical approval, and IO therapies have so far only shown modest improvement over these figures (3, 38). Early integration of clinically relevant preclinical models can impact drug success rates by enabling more informed decisions during drug discovery, while broad patient-derived model cohorts tested early in the pipeline facilitate biomarker discovery and patient stratification for clinical trials (39). As next-generation oncology therapeutics grow increasingly complex – progressing from monospecific antibodies to bispecifics and trispecifics (40–42), CAR-T/NK cell therapies with multimodal targeting (43), and multifunctional agents engaging both innate and adaptive immunity (44) – the need for advanced, human model systems that can replicate this complexity becomes critical.

Here, multiple cancer indications and therapeutic modalities were validated for advanced *in vitro* 3D IO drug testing, using two complementary methods: an organoid-based reconstituted assay and a native EVPT assay. Depending on drug development stage, drug mechanism of action, and research question, these models offer high patient relevance for evaluating clinical-stage candidates in HCI assays (**Table 1**).

**Table 1:** Comparison of advantages and limitations across 3D IO platforms.

Feature		Reconstituted TME	*Ex vivo*
Assay contents	+	Fully controlled co-culture contents, e.g. Tumor-Effector ratio Multiple PBMC donors tested in same assay Immune cells & fibroblast triple co-cultures	Complete and naïve patient-specific TME Co-culture with autologous PBMCs for TME enrichment TME heterogeneity across condition
	–	Need to control for allogenic response	Biopsy or resections material represent focal tumor
Biobank material	+	Pre-banked model selection TAA selection based on RNAseq, CNV, Proteomics Mutational background/WES available Histopathological subtyping Resistance modes available	
	–		Limited preselection of subtype, resistance, mutation background Dependent on availability of fresh tissue
Sample characterization	+	Compatible with deep biomarker characterization HLA-typing	Flow cytometry-based biomarker characterization of fresh samples (prospective) Representative pathology characterization possible (retrospective)
	–		Limited sample quantity requires prioritization of assay *vs* characterization
Assay Methods	+	Label-free HCI assay Biochemical assays (LDH, ATP, etc) Label-dependent analyses (engineered models) Cytokine analyses possible (supernatant)	Label-free HCI assay Flow Cytometry assay and analyses Cytokine analyses possible (supernatant)
	–		
Assay capacity	+	Fully scalable, reproducible assay setup possible (incl replicates) Biobanked material for bespoke assays Flexible setup Parallel biomarker assays possible	Technical replicates included in HCI (n=8) Combination with auxiliary assays possible
	–		Limited conditions/lower throughput
Therapy modality	+	Antibodies and antibody-derivatives, cellular therapies, small and large molecules, viruses, combination therapies	Same as reconstituted
	–		
Application	+	Medium-large efficacy screening (multi-compound and/or -patient) Mechanism of Action studies Matched PDX in vivo follow-up possible	Lead molecule testing/Late stage preclinical Relevant patient cohort selection
	–		

The key advantages, limitations and differences between the platforms (and any similar organoid- or *ex vivo*-based platform) are highlighted. In summary, minimal complexity in a reconstituted assay allows for MoA studies utilizing fully characterized models with known genetical features. Due to scalability of organoid cultures, it allows large-throughput experiments testing many compounds on many models. ICI testing is possible, although the limited complexity may not always provide the full picture. The full complexity of *ex vivo* drug screening on primary tumors allows drug testing in an autologous setting, but is limited in the number of samples and compounds to be tested, as well as in characterization of the tumors used. The *ex vivo* setting is therefore best suited for testing ICIs and other therapeutics that require full complexity, as well as for drugs further down the pipeline to provide most relevant information on patient-to-patient response variation.

The underlying automated 384-well HCI-based drug screening format of both platforms is suited for large throughput screens with high reproducibility for commercial use. Compared to conventional high-throughput IO screening, such as 2D co-cultures where direct contact between effector and target cells is guaranteed, the 3D environment of the assay requires migration and infiltration of immune cells through the matrix and into the multicellular objects providing a more clinically translatable testing of IO therapeutics. Multiparametric read-outs by imaging without dissociation in combination with multiple time-point replicates, allow for the specific probing of these migration and infiltration processes in addition to morphological read-outs of tumor killing, thereby addressing multiple important steps in the mode of action of the tested therapy.

Key attributes of valuable *in vitro* screening platforms include clinical translatability, model characterization, assay reproducibility and scalability; all of which will be discussed below.

Organoids derived from tumor stem cells have demonstrated strong translational value, showing high correlation with clinical responses in retrospective studies across chemotherapy, radiotherapy, and targeted therapy (18–21). Patient-derived xenograft organoids (PDXOs) also preserve genetic, transcriptomic, and drug-response profiles of their parental models, comparable to preclinical “gold standards” (11, 15) and pave the way for a smooth *in vitro* to *in vivo* transition. In IO testing, both tumor and immune cells influence IO therapeutic efficacy, but despite slight donor-to-donor variability, tumor cell biology plays a dominant role (**Figure 2**). Allogenic organoid-based co-culture systems will consequently be valuable tools for preclinically predicting patient outcomes and supporting “preclinical trials in a dish”.

Immune cell activation (as measured by cytokine profiling) did not always correlate with increased tumor cell killing, underscoring the value and need for a high-content imaging-based analysis pipeline. While IFN-γ has been well studied as a predictive marker for immune checkpoint therapy responses across different cancer indications and outperforms other expression signatures in metastatic melanoma, IFN-γ alone cannot fully predict a patient’s response (45–47), corroborating our findings. Whether IFN-γ can be used as prognostic marker for other immunotherapies such as BiTEs is not yet firmly established, but is supported in combination with other cytokines and inflammatory markers (48, 49). Therefore, to fully comprehend tumor-specific responses to existing and newly developed compounds, we recommend testing drug responses in multiple patient-derived models using both cytokine and viability assays.

The commercial availability of large-sized, deeply characterized biobanks (including eg WES, RNAseq, proteomic and histological characterizations (15)) allow both for the selection of models of interest, based on antigen expression, genetic features, drug target, or resistance profile of interest, as for biomarker identification upon testing drugs such as bispecific T cell engagers and cellular therapies like CAR T cells on large organoid panels. Testing of AMG910, a Claudin 18.2 targeting BiTE, showed the consistency between RNA levels, protein surface levels and immune cell activation, infiltration and immune-mediated killing. Moreover, both the individual models shown in **Figure 2e** and the >100 model IO screen presented in **Figure 2f** illustrates the assay scalability, value, and window of responses one can identify across a patient population. The observed degree of sensitivities was likely influenced by several factors, including variability in tumor antigen expression, the presence of immune-suppressive mechanisms, and morphological and growth characteristics of the organoids, all determined by the underlying genetic differences between models. Some PBMC donor-to-donor variations are observed in a subset of organoid models, it is therefore recommended to always test multiple donors per model if possible to cover potential variation of measured sensitivity. Overall, consistency between multi-donor testing illustrated assay reproducibility, and testing immunotherapies across a diverse panel of organoid models therefore provides a representative assessment of potential drug responses within a patient population.

For assays reflecting native tumor complexity, the EVPT assay offers testing in mechanically dissociated patient tumors. Though this approach partially compromises the original tissue architecture such as macro-level spatial organization as well as multiple distinct areas in tumor foci where the local TME periphery varies in terms of cell-cell interactions and gradients, it favors reproducible distribution of the entire tumor complexity across technical replicates, retaining multicellular tumor clusters with autologous immune cells, fibroblasts, and stromal elements. The object size in the plate-based assay is no more than 200 µm, which is sufficiently small for full oxygenation and nutrient accessibility (50).

All tumor resection tissues tested here were procured through commercial tissue providers. The associated clinical outcome of these specific patients was unavailable, nor were they part of a dedicated clinical trial and most received first line therapies. Still, across our tested NSCLC sample cohort, EVPT testing identifies 20 – 25% of samples responding to immune checkpoint inhibitor (ICI), which is in the same range as reported clinical outcomes in NSCLC, and seemingly distinguishing responders from non-responders (36). Comparable clinical correlations have been reported in high-grade serous ovarian cancer (30), and MoA discovery for PD-1/PD-L1 sensitivity has been demonstrated in alternative *ex vivo* assays (51) in similar contexts (28, 29). Notably, NSCLC tissues treated with the pan-immunostimulatory superantigen SEA exhibited ~50% response (**Figure 4g**), far exceeding clinical responses to ICI treatment (36) suggesting potential benefit for patients unresponsive to conventional ICIs.

Alike the organoid platform, EVPT is also suitable for testing non-IO therapeutics, like antibody drug conjugates, peptides, small molecules, lipid nanoparticles, and standard-of-care regimens. Here, the value is especially high for indications with limited availability of robust and translational preclinical screening models, such as glioblastoma (52) or prostate cancer (53). Despite constraints of tissue availability and upfront sample characterization, EVPT supports multi-modal characterization (flow cytometry, NGS, IHC) and cytokine/immunofluorescence analyses, allowing biomarker validation studies.

While both HCI assays have scale to allow for testing multiple doses, timepoints, or combinations, the reconstituted organoid assay provides greater scalability, enabling medium- to high-throughput testing, reproducibility assessment, and iterative experimentation – ideal for early-phase drug development research. In contrast, EVPT assays are better suited for near-IND lead candidates, with an assay capacity that enables ~15 treatment conditions per tumor resection. Depending on sample composition and complexity, and due to the relatively large heterogeneity of the source material, the EVPT assay window can be modest, with a non-significant median drop in volume or object count of 10-30%. The limited assay runtime of 5–7 days, which cannot be extended due to detrimental effects on the assay performance related to either sample dependent impaired cell survival or cell overgrowth, may not always be sufficient to present a substantial ICI response, especially for samples with low TIL presence or when TILs may not be sufficiently potent to kill most tumor cells. In those samples, even a small response should be considered relevant. To further support a more robust interpretation of drug performance, each treatment condition consists of octuplicate technical replicates.

In summary, the organoid-based reconstituted assay excels in availability, characterization, reproducibility, and scalability, whereas EVPT offers unmatched translational relevance and preservation of the autologous microenvironment (**Table 1**). HCI-technology outperforms biochemical readouts like ATP- or NAD(P)H-based viability assays when sample complexity increases by measuring differential responses between cellular subsets. This is especially relevant for IO assays; allowing labeled immune and tumor cell tracking in the reconstituted assay, or mitigating the relatively high basal level of cell death and associated readout noise observed in *ex vivo* samples. In addition, due to the small tissue requirements per well of the 384-well plate setup, still a relatively large number of conditions can be tested compared to more traditional *ex vivo* assays, such as precision cut tumor slices (54). While various methodologies discussed above have unique properties for supporting drug evaluation in pre- or near-clinical environments, the presented IO platforms here present valuable tools for supporting current multimodal and multi-target drug discovery pipelines. In addition, regulatory changes, including the FDA Modernization Act 2.0 (US FDA, 2022), have removed the animal testing mandate for safety and efficacy evaluation. As the FDA roadmap prioritizes validated non-animal methodologies (US FDA Roadmap, 2021), advanced *in vitro* platforms like these are well positioned to accelerate the transition and expand their role in oncology drug development.

## Materials and methods

### 384 well screening assay

Drug screening was performed as described before (23). Briefly, clear bottom 384-wells plates (µclear, Greiner Bio-One 781091) were used. Per well, 12 µl (reconstituted) or 14 µl (EVPT) of a 0.5 mg/ml collagen (Corning Collagen I HC, rat tail, Cat#354249) and 60% Matrigel (Corning, Cat# 356231) with cell material mix was dispensed using a CyBio Felix automated liquid handling robot (Analytik Jena, Jena, Germany). Upon 30 minutes gelation at 37 °C, medium was added on top of the gel in each well to a total volume of 54 µl. For the reconstituted assay the medium used contains RPMI-1640 (21875034, Gibco) with human AB serum (H4522, Sigma Aldrich), Penicillin/Streptomycin (15140122, Gibco), 1% Hepes (15630056, Gibco), 10 µg/ml IL-2 (200-02, Peptrotech). For EVPT the medium used contains Advanced DMEM F12 (Gibco, cat# 12634028), 2 mM Glutamax (Gibco, cat# 35050038), 100 U/mL PenStrep (Gibco, cat# 15140122), 0.01 M HEPES (Gibco, cat# 15630080), 1x antimycotic solution (Sigma-Aldrich, cat# A5955), 1x B27 (Gibco, cat# 17504001), 10 ng/mL Heregulin (ImmunoTools, cat# 11343045), 10 ng/mL hFGF-b (PeproTech, cat# 100-18B), 0.01 mM Y27632 (SelleckChem, cat# S1049) and 10 ng/mL hEGF (PeproTech, cat# AF-100-15) Therapeutics, including control and reference compounds, were mixed with the medium in v-bottom 96-wells plates as 10X stocks before adding these in 6 µl to the 384-wells plates with solidified gel. At the experimental endpoint, the samples were fixed and stained with Hoechst 33258 and Rhodamine-phalloidin to visualize nuclei and F-actin (actin cytoskeleton) respectively as previously described (55).

### Reconstituted assay

Organoid models were established and maintained as described (15, 18). WES and RNAseq data from organoids were collected and analyzed as previously described (15). Size filtered organoids were seeded in quadruplicate technical replicates in 3D hydrogel in 384-well plates as described before (23) and incubated for approximately 72 hours before co-cultures were initiated. Human PBMCs were obtained from buffy coats from healthy volunteers obtained from the Dutch blood bank (Sanquin, Amsterdam, The Netherlands), isolated by density gradient using Ficoll Paque (GE Healthcare, Uppsala, Sweden) and cryopreserved until experiment execution. Thawed PBMCs were stained with cell tracker (CellTracker Green CMFDA Dye, Invitrogen Cat #C2925) and added on top of the 3D hydrogel with 5:1-10:1 E:T ratio. This E:T ratio was estimated based on organoid size and number vs PBMC numbers. Of the 100 model screen, 70 models were screened with multiple donors to test for consistency. Co-cultures were treated with Staphylococcal enterotoxin A (SEA) (Sigma/Merck, Cat #S9399) as a positive control for the assay as it strongly activates human lymphocytes and promotes tumoroids to be infiltrated by the immune cells. Test treatments included bispecific antibodies targeting EGFR and CD3 (0.2 ng/mL-2 µg/ml) (Creative Biolabs- #CBL-BilE003-11) or BSA and CD3 control (2 µg/ml) (Creative Biolabs, Cat #BSFV-C001), Claudin 18.2 targeting BiTE AMG910 (1 ng/mL-10 µg/mL) (MCE, Cat #HY-P99350) or Ultra-LEAF™ Purified Human IgG1 Isotype Ctrl (10 µg/mL) (Biolegend, Cat #403501), EGFRscFv-CD28-CD3ζ CAR T-cells and Non transduced T cells (both ProMab Biotechnologies). CAR-T and Non transduced T cells were expanded by the supplier for 9–13 days using CD3/CD28 beads (Gibco) at a 1:1 cell to bead ratio in CAR-T cell medium supplemented with FBS and kept at a culture density of 0.5-1e6 cells/mL until day 9-13. After 48- and 96-144-hours, supernatants were collected for cytokine analysis and plates were fixed and stained for imaging and analysis.

### *Ex vivo* patient tissue assay

Ultra-fresh cancer patient tumor material was obtained through commercial tissue providers. Resections were from treatment-naïve patients, or from patients who were not treated for a minimum of six months, to prevent including samples with poor cell viability. All materials were shipped on ice emerged in T-store (Life Science Production) media supplemented with antibiotic and antifungal preservatives and received within 24 hours of collection to minimize tissue decay and maximize cell stability and viability. Upon receipt, sample size and composition of each tissue was evaluated, necrotic areas, large fat deposits, and fibrous sections were removed. Remaining tumor tissues were cleaned and washed in PBS and media and cut into smaller fragments using scalpel and scissors. Next, they were mechanically sheared using pipette tips until an easy-to-pipet cell suspension was obtained. In case of significant erythrocytes presence, these were removed through lysis. Extended shearing to obtain a heterogenous mix of cell material from the TME containing a range of cells from single cells to multicellular clusters (<200 µm) was performed using either continued manual dissection using scissors and scalpels or using the gentleMACS Octo Dissociator (Miltenyi Biotec, Cat #130-134-029). Great care is taken in not overprocessing the sample, and for HCI preparation the aim is to preserve clusters as much as possible over a fully dissociated sample. Next, the heterogenous tumor-TME mix was taken up in hydrogel and 50–300 multicellular clusters >50 µm and <200 µm in diameter were seeded per well into 384-well plates as described above. Tissues were exposed in octuplicate technical replicates to solvent controls PBS (negative controls), Staurosporin 1 µM (positive control for cell killing) (MedChem Express, Cat #HY-15141), and SEA (positive control for immune-mediated killing). Therapeutic antibodies used include PD1 inhibitor pembrolizumab (20 µg/ml) (Keytruda), CTLA-4 inhibitor ipilimumab (20 µg/ml) (Yervoy), bispecific antibodies targeting EGFR and CD3 (20 µg/ml) (Creative Biolabs, Cat #CBL-BilE003-11) or BSA and CD3 control (20 µg/ml) (Creative Biolabs, Cat#BSFV-C001), EGFR-targeting Cetuximab (20 µg/ml) (Erbitux), and anonymized antibody compound cmpdX. The cultures in 384-well plates were incubated at 37 °C and exposed to compounds for 5–10 days, before fixing and staining for HCI analysis. Samples found to have cumulative volume <0.01 mm^3^ of clusters were QC-failed and excluded from further analyses. Sample response was defined by a stepwise reduction in tumor volume in categories of 10-30%, 31-50% 51-70%, and >71%.

### In-gel IF staining

For staining of activated immune cells, Alexa Fluor^®^ 647 anti-human CD69 Antibody (Biolegend, Cat #310918) was incubated 6 hours in culture medium prior to fixation. Cultures were fixed with 4% PFA at room temperature, washed with PBS, followed by staining of the nuclei and actin cytoskeleton as described above. For staining of CD45, CD3 and EpCAM (CD326), cultures were fixed and washed, permeabilized at room temperature (10% BSA (Miltenyi, Cat #130-091-376), 10% Triton X-100 (Merck, Cat #T8787) in PBS) and subsequently incubated in blocking buffer (2% BSA and 10% Triton X-100 in PBS). Next, cells were incubated with fluorophore-conjugated primary antibodies against EpCAM-APC (ThermoFisher, Cat #MA5-38715), CD3-FITC (BioLegend, Cat #300406), CD45-FITC (Thermo Fisher Scientific, Cat #51-0459-42) diluted in antibody dilution buffer (10% BSA, 10% Triton X-100 in PBS). Samples were washed with TBP (10% BSA, 10% Triton X-100 in PBS), washed in PBS and counterstained for nuclei and actin cytoskeleton as described above.

### High content imaging and image analyses

Imaging of the plates was performed using a ImageXpress Micro XLS (Molecular Devices, Wokingham, UK) as previously described (22). Briefly, each well in a 384-wells plate was captured using a 4x objective lens, with a z-step size of 20-50 µm to acquire z-stacks of each individual channel for nuclei, actin, and celltracker. The number of sections per well ranged between 20 sections to 80 sections, to capture the entire height of the gel per well. Confocal images were captured using an ImageXpress Micro Confocal (Molecular Devices) using a 20x 0.75NA objective and processed in ImageJ. Captured images are stored on a central data server, accessible by the Crown Bioscience Ominer^®^ image analysis platform integrated into the KNIME Analytics Platform (http://knime.com/), as described (23). Briefly, the platform identifies individual object structures (nuclei and cytoskeleton) per well, and their relative positions. A rigorous quality control process ensures the segmentation output images represent the input images, next to a quality control of the input data as well, and exclude individual well errors that result from gel instability, staining perturbations and low fluorescent intensity of labels, or high levels of cell debris. Typically, <4% of wells (equivalent to 10 well out of a total of 260 for a full plate) are excluded from the assay from a combination of these factors. Next, output measurements include per-object measurements of nucleus shape and number, tumoroid number and shape, and pre-labeled cells, aggregated per well and technical replicates. Data were then checked for consistency within control treatments, absence of edge effects, and consistency between replicates. Large object or tumoroid size reflects the cumulative measured area of segmented organotypic structures that is defined by the presence of cell nuclei within an intact actin cytoskeleton structure of multicellular objects and measured throughout the image-stack of each well. Object size can be shown as cumulative, or averages across all objects in the same well. Object count depicts the number of individual objects measured in a segmentation mask. The single cell count is the number of cells measured, with a maximum object size to restrict measurements to individual cells. The infiltration measures pre-labeled cells that are present within a multicellular object.

### Luciferase assay

To measure organoid viability of the reporter LU5178-lucg model, the Steady-Glo kit according to manufacturer’s protocol was used (Promega, Cat #E2510). T cells were isolated with Pan T Cell Isolation Kit, human (Miltenyi, Cat #130-096-535) according to the manufacturer’s instruction and pre-activated with 100 ng/mL SEB. Co-cultures were executed in suspension at 1:1 E:T ratio and analyzed after 120h.

### Cytokine assay

Supernatants of cultures were analyzed for cytokine levels via multiplex using meso scale discovery (MSD) kits (V-PLEX Proinflammatory Panel 1 Human Kit) or no-wash Immunoassay for IFN-γ detection via Lumit kit (Promega, Cat #W6040). Manufacturers procedures were followed for cytokine detection.

### Flow cytometry

For FACS of organoids, size filtered organoids were seeded in Matrigel droplets in 6-well plates and incubated for approximately 72 hours. Organoids were then harvested, washed to remove residual matrix and dissociated into a single cells using TrypLE (Invitrogen, Cat #12604013). Cells were fixed for 15 minutes with 4%PFA at room temperature, and washed twice with PBS. All staining and washing steps were performed in 100 µL FACS buffer (1% BSA (Miltenyi, Cat #130-091-376) in PBS) with centrifugation steps carried out at 1500 rpm for 5 min. 100.000 cells per sample were stained in with 10 µg/mL AMG910 (MCE, Cat #HY-P99350) for 15 minutes at room temperature and washed twice. Next, cells were incubated with Goat anti-Human IgG (H+L) Cross-Adsorbed Secondary Antibody, Alexa Fluor™ 647 (Thermo, Cat #A-21445) for 30 minutes at 4 °C and washed twice and resuspended in 100 µL FACS buffer for acquisition.

For FACS of EVPT samples, heterogeneous mixtures of tumor clusters and immune cells were isolated from surgical cancer resection specimens as described above and dissociated into a single-cell suspension using a double-tip mechanical shearing method in cold AdDMEM4x+ (Advanced DMEM (Life Technologies\12634010) supplemented with 1% GlutaMAX (Thermo Fisher Scientific Cat #35050038), 1% HEPES (Thermo Fisher Scientific Cat #15630056), and 1% BSA (Miltenyi, Cat#130-091-376) and processed for baseline flow cytometry analysis (0 hours). The remaining material was embedded in Matrigel droplets and cultured in 6-well plates for 24 or 144 hours. Cell material was harvested, washed to remove residual matrix and dissociated into single cells using the double-tip shearing method. All staining procedures were performed in staining buffer (0.5% BSA in PBS) in the dark at 4 °C, with centrifugation steps carried out at 1500 rpm for 5 min unless otherwise specified. First, 1.000.000 viable cells per sample were stained with eBioscience™ Fixable Viability Dye eFluor™ 780 (Thermo Fisher Scientific, Cat #65-0865-18) for 30 min, and washed. Cells were then incubated with Brilliant Stain Buffer (BD Biosciences, Cat #563794) and Fc receptor blocking reagent (Miltenyi Biotec, 130-059-901). Next, cells were stained for CD45 (BD Biosciences, Cat #564105), CD3 (BioLegend, Cat #6198), CD8 (BioLegend, Cat #6247), CD14 (BioLegend, Cat #7366), CD56 (BD Biosciences, Cat #740979), CD25 (BD Biosciences, Cat #565096), and CD127 (BD Biosciences, Cat #560822) and washed. Finally, cells were fixed in PFA at room temperature, washed, and resuspended in PBS. Compensation controls were prepared using UltraComp eBeads (Invitrogen, Cat #01-3333-42).

Samples were acquired using Novocyte Quanteon (Agilent) and analyzed using NovoExpress or FlowJo.

### Single and multiplex IHC and imaging with quantification

Downstream analysis was performed on processed tissue, despite the loss of tissue architecture and the detrimental effects of tissue shearing and processing, as this approach was more representative of the sample in its entirety rather than focusing on a fragment of the tumor prior to processing. After tissue processing (tumor mincing and shearing), a portion of the tumor sample was collected prior to treatment, for fixation in 4% PFA and subsequent washing in PBS prior to transfer to 70% Ethanol. Samples were stored at 4 °C until embedding as FFPE blocks following standard tissue processing protocols, including dehydration, clearing, and wax immersion. FFPE blocks were sectioned at 4 µm thickness using a semi-automated rotary microtome (Leica HistoCore MULTICUT) and tissue slices were adhered to superfrost glass slides. These slides were baked at 60 °C for 60 min. The following steps were performed using the Leica Bond RX autostainer; Dewaxing and rehydration using Bond™ Dewax solution and graded ethanol; Antigen retrieval using Bond™ Epitope Retrieval Solutions (Bond™ Epitope Retrieval Solution 1 (Leica, Cat. No. AR9961) and 2 (Leica, Cat. No, AR9640) at 100 °C; Blocking endogenous peroxidase activity with Peroxide Block; Primary antibody incubation specific to the panel, followed by washing steps; Visualization using Leica Bond DAB detection kit for single chromogen IHC or Leica Bond DAB detection kit with TSA Opal fluorophores (520/570/650) for multiplex IHC. Counterstaining with Hematoxylin or DAPI and mounting with SlowFade™ Gold antifade reagent (Life Technologies, Cat. No. S36938). All stained sections were scanned using NanoZoomer S60 imaging systems, producing high-resolution images (40x magnification). Image analysis and quantitation were conducted using HALO™ Image Analysis platform, with necrotic regions excluded. Phenotypic quantifications including %EPCAM+ cells, %α-SMA+ cells, and CD45^+^ cells. Primary antibodies: EpCAM (CST Cat #14452), aSMA (Abcam, Cat #ab5694); CD45 (CST, Cat #13917), Pan-CK (CSK, Cat #4545S), CD3 (Thermo, Cat #MA5-14524), CD4 (Abcam, Cat #ab133616), CD8 (Thermo, Cat #MA5-14548), CD68 (Thermo, Cat #MA5-13324), CD80 (Abcam, Cat #ab134120), CD163 (Abcam, Cat #ab182422), FoxP3 (CST, Cat #98377), PD-L1 (CST, Cat #13684), PD1 (CST, Cat #86163), CTLA-4 (CST, Cat #96399). Followed by secondary detection (Leica, Cat #DS9800) and Akoya Opal Fluorescence: Opal 520 (AKOYA, Cat #FP1487001KT), Opal 570 (AKOYA, Cat #FP1488001KT), Opal 650 (AKOYA, Cat #FP1495001KT). Counter stain: DAPI (abcam, Cat #ab228549).

### Statistics

The reconstituted assay used quadruplicate technical replicates, primary tissue EVPT uses octuplicate technical replicates. All data were presented as mean ± SD. Statistical analysis was performed in Graphpad Prism 10.5.00. For datasets where two or more variables are present, a two-way ANOVA was performed, followed by multiple comparisons using Tukey’s methodology. This applies to **Figures 2b**, **3b**, **4b**, **5b, 5d**. For **Figure 3c** a two-way ANOVA was performed against IgG control, followed by multiple comparisons using Dunnett methodology. For datasets where one variable is present, a one-way ANOVA was performed, followed by multiple comparisons using Tukey’s methodology. This applies to **Figures 2c**, **3a, e**. Unpaired t-test comparing two means was used for **Figure 3b**. **** is P<0.0001, *** is P<0.005, ** is P<0.01, * is P<0.05. **Figure 2d** shows coefficient of variation (CV%), defined as the ratio of the standard deviation to the mean.

## Data Availability

The raw data supporting the conclusions of this article will be made available by the authors, without undue reservation.
